# Supplementary material for: Buprenorphine Increases HIV-1 Infection In Vitro but Does Not Reactivate HIV-1 from Latency
Source: Viruses. 2021 Jul 27;13(8):1472. doi: 10.3390/v13081472 (PMC8402857; doi:10.3390/v13081472)
Supplement: Supplementary file 1 [file viruses-13-01472-s001.zip › viruses-1219772-supplementary.pdf]

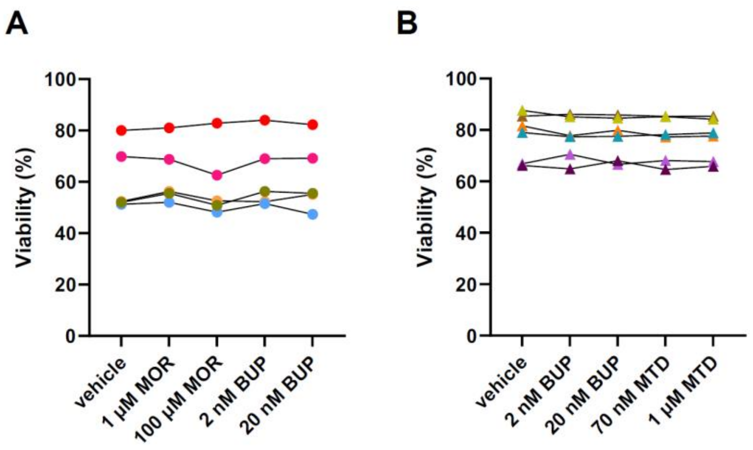

Figure S1. Determination of viability for different drugs and concentrations. **(A)** We treated PBMCs derived from 5 different donors (represented with different colors) for 72h with R10 medium supplemented with 4 µg/ml PHA, 50 U/ml IL-2 and either morphine (MOR) (1 or 100 µM) or buprenorphine (BUP) (2 or 20 nM). The vehicle control contained 1.25% v/v of methanol, matching the concentration of methanol present in the cultures with 100 µM MOR. We determined cell viability using trypan-blue staining or Guava Muse® analyzer. The viability in the vehicle control had a mean of 61.12% (range: 51.3-80.0%, n=5) **(B)** We determined cell viability in PBMCs derived from 6 different donors. PBMCs were cultured for 72h with R10 medium supplemented with 4 µg/ml PHA, 50 U/ml IL-2 and either buprenorphine (2 or 20 nM) or methadone (MTD) (70 nM or 1 µM). Because buprenorphine and methadone stock solutions were prepared in water, the vehicle controls in these experiments did not contain 1.25% v/v methanol. The mean viability in the vehicle-control treated cultures was 77.78% (range: 66.2-87.6%, n=6).

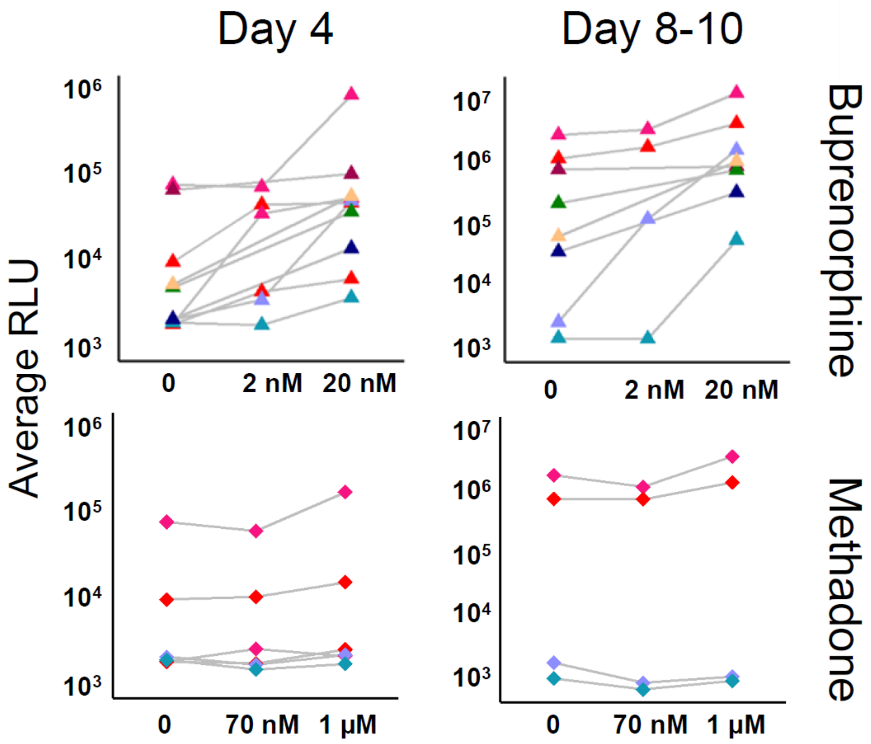

Figure S2. Comparison of methadone and buprenorphine on HIV-1 propagation in longer-term PBMC cultures. These data are the same as in Figure 4B, but showing the raw values. PBMC were incubated with opioids for 72h, infected and washed, then the opioid drugs were added back to the culture media. Aliquots were taken at days 4 (left panel) or 8-10 (right panel), and HIV-1 replication was measured by quantifying nanoluciferase activity in the supernatants. Chemiluminescence signals were quantified using a luminometer. The results shown are for a MOI of 0.5. Each donor is represented by a different color. We did between 4 and 12 technical replicates per condition; each symbol represents the average relative light units (RLU) of these replicates for a given donor and treatment.

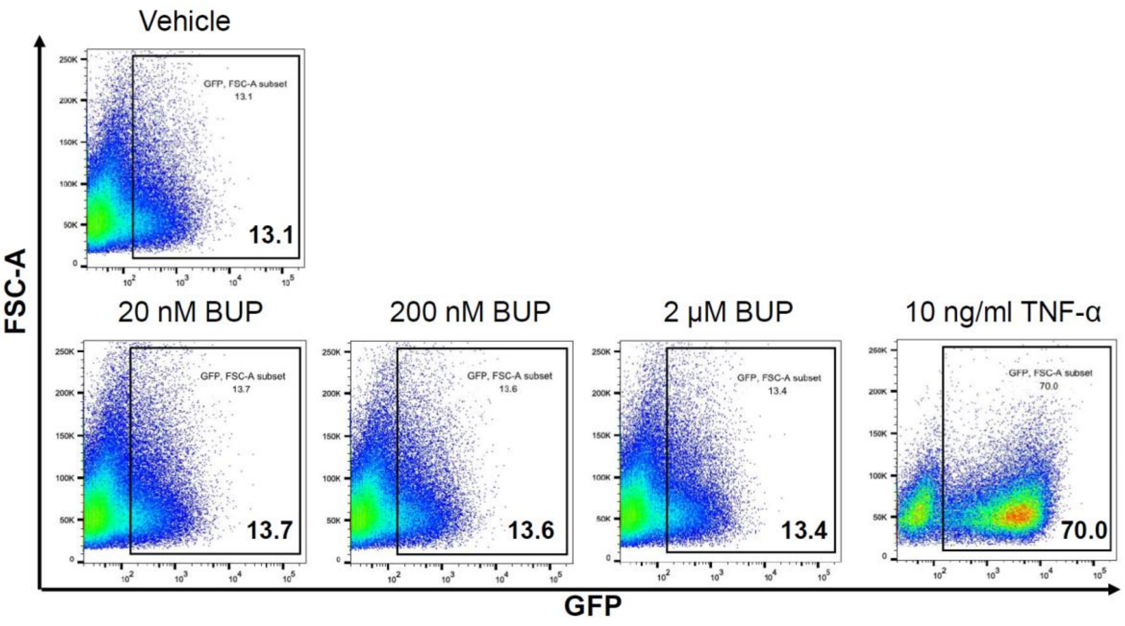

Figure S3. Effect of buprenorphine on HIV-1 reactivation in the HIV-1 latently infected T cell line J-Lat A7. J-Lat cells (clone A7) were treated for 72h with vehicle (R10 medium alone) or buprenorphine (20 nM, 200 nM or 2 μM). TNF-α (10 ng/ml) treatment was included as a positive control for reactivation. GFP expression was evaluated in live (NearInfraRed^{negative}) cells by flow cytometry. Y-axis, scatter signal; X-axis, GFP fluorescence. This experiment was done with 2 technical replicates and only the dot plots from one of the duplicates are shown. Numbers in the lower right corners denote % GFP+ cells. Cell viability (% NIR^{negative}) was 96.5%, 96.2% and 76.4% for vehicle-, 2 μM BUP- and TNF-α-treated cultures, respectively.
